# Meta-analysis of nifedipine and enalapril combination therapy for hypertensive patients with coronary heart disease: A systematic review and meta-analysis

**DOI:** 10.5937/jomb0-52387

**Published:** 2025-01-24

**Authors:** Wang Kun, Ma Wenchao, Sun Leina, Su Fangcheng

**Affiliations:** 1 Weifang People's Hospital, Department of Respiratory and Critical Care Medicine, Weifang, China; 2 Weifang People's Hospital, Department of Emergency, Weifang, China

**Keywords:** nifedipine, enalapril, hypertension, coronary heart disease, meta analysis, nifedipin, enalapril, hipertenzija, koronarna bolest srca, meta analiza

## Abstract

**Background:**

Numerous clinical studies have demonstrated that the therapeutic efficacy of combining nifedipine and enalapril in treating hypertension with coronary heart disease surpasses that of nifedipine as a stand-alone treatment. However, the current evidence is not yet sufficient due to limitations in the sample size, and further validation is needed. To analyze and assess the clinical impact of nifedipine combined with enalapril on hypertension complicated with coronary heart disease, and provide evidence for rational drug use in clinic.

**Methods:**

We employed a revised version of the Jadad scale to assess the quality of the research literature following a rigorous screening process. The statistical analysis was performed utilizing the software RevMan 5.4.1 for data analysis. Heterogeneity in the studies was evaluated based on the results of the Q test (P value), and the OR value of the combined effect was calculated using either the model with fixed effects or the one with random effects, with the results presented in a forest plot. Furthermore, a sensitivity analysis was conducted by excluding articles with the highest impact, and potential bias in publication was assessed through the utilization of a funnel plot.

**Results:**

A total of 183 articles were initially identified, and after a comprehensive review, 14 clinical randomized controlled trials were chosen for analysis. The meta-analysis findings revealed that the trial group displayed a significantly higher overall effectiveness rate compared to the control group (OR=3.47, 95%CI 2.40-5.03). Additionally, the trial group exhibited a more pronounced reduction in blood pressure and greater enhancement in cardiac function (OR=5.55, 95%CI). Conversely, the control group had a lower occurrence of ischemic events compared to the trial group (OR=0.35, 95%CI 0.24-0.50). Sensitivity analysis confirmed the stability and reliability of the combined effect size results (OR=3.91, 95%CI 2.51-6.09, P<0.00001). However, based on an assessment using funnel plot results suggested potential bias in publication.

**Conclusions:**

The combined administration of nifedipine and enalapril exhibits enhanced effectiveness compared to the sole use of nifedipine in individuals diagnosed with hypertension and coronary heart disease, rendering it a valuable alternative for clinical application.

## Abstract (sr)

**Uvod:**

Brojne kliničke studije su pokazale da terapijska efikasnost kombinovanja nifedipina i enalaprila u lečenju hipertenzije sa koronarnom bolešću srca prevazilazi efikasnost nifedipina kao samostalnog lečenja. Međutim, trenutni dokazi još uvek nisu dovoljni zbog ograničenja u veličini uzorka i potrebna je dalja validacija. Potrebno je analizirati i proceniti klinički uticaj nifedipina u kombinaciji sa enala - prilom na hipertenziju komplikovanu koronarnom bolešću i pružiti dokaze za racionalnu upotrebu lekova u klinici.

**Metode:**

Koristili smo revidiranu verziju Jadad skale da procenimo kvalitet istraživačke literature nakon rigoroznog procesa skrininga. Statistička analiza je izvršena korišćenjem softvera RevMan 5.4.1 za analizu podataka. Heterogenost u studijama je procenjena na osnovu rezultata K testa (P vrednost), a OR vrednost kombinovanog efekta je izračunata korišćenjem ili modela sa fiksnim efektima ili modela sa slučajnim efektima, sa rezultatima prikazanim u brojnim studijama. Štaviše, sprovedena je analiza osetljivosti isključivanjem članaka sa najvećim uticajem, a potencijalna pristrasnost u objavljivanju procenjena je korišćenjem dijagrama toka.

**Rezultati:**

Prvobitno je identifikovano ukupno 183 članka, a nakon sveobuhvatnog pregleda, za analizu je izabrano 14 kliničkih randomizovanih kontrolisanih studija. Nalazi meta-analize su otkrili da je ispitivana grupa pokazala značajno veću ukupnu stopu efikasnosti u poređenju sa kontrolnom grupom (OR=3,47, 95% CI 2,40-5,03). Pored toga, ispitivana grupa je pokazala izraženije smanjenje krvnog pritiska i veće poboljšanje srčane funkcije (OR=5,55, 95%CI). Suprotno tome, kontrolna grupa je imala manju pojavu ishemijskih događaja u poređenju sa probnom grupom (OR=0,35, 95% CI 0,24-0,50). Analiza osetljivosti je potvrdila stabilnost i pouzdanost rezultata kombinovanog efekta (OR=3,91, 95%CI 2,51-6,09, P<0,00001). Međutim, na osnovu procene korišćenjem dijagrama toka, rezultati sugerišu potencijalnu pristrasnost u publikaciji.

**Zaključak:**

Kombinovana primena nifedipina i enalaprila pokazuje povećanu efikasnost u poređenju sa jedinom upotrebom nifedipina kod osoba sa dijagnozom hipertenzije i koronarne bolesti srca, što ga čini vrednom alternativom za kliničku primenu.

## Introduction

Cardiovascular disease is heavily influenced by hypertension, which is considered one of the most significant risk factors [1]. The risk of developing cardiovascular disease is positively associated with blood pressure levels. In recent years, the prevalence of hypertension comorbid with coronary heart disease has been rising among middle-aged and elderly individuals due to changes in lifestyle. The presence of hypertension exacerbates the progression of atherosclerosis, particularly in the coronary arteries, cerebral arteries, renal arteries, and peripheral arteries, thus leading to multi-organ dysfunction [2]. The concurrent utilization of nifedipine and enalapril demonstrates heightened efficacy in individuals with hypertension and coronary heart disease, presenting a valuable clinical alternative when compared to the exclusive administration of nifedipine. The complications of hypertension and coronary heart disease include angina pectoris, myocardial infarction, sudden death, and eventually heart failure, posing a severe threat to the well-being and survival of patients [3] [4] [5]. Consequently, finding effective strategies to concurrently reduce blood pressure and coronary artery plaque in patients with hypertension and coronary heart disease becomes crucial for improving myocardial blood supply and ultimately benefiting patients’ health.

Nifedipine, a calcium channel blocker, promotes smooth muscle relaxation in blood vessels by reducing intracellular calcium ion levels, resulting in decreased arterial pressure. Due to its gentle and long-lasting hypotensive effect, it has gained wide-spread usage [6]. Enalapril works by blocking angiotensin converting enzyme (ACE), which leads to lower levels of angiotensin II being produced.. This mechanism effectively blocks the vasoconstriction, water and sodium retention, and subsequent blood pressure elevation caused by angiotensin II. Moreover, enalapril has the ability to enhance arterial elasticity and lower the dynamic arterial stiffness index in individuals with hypertension, effectively suppressing vasoconstriction and ultimately exerting a sustained antihypertensive effect [7].

Numerous clinical studies [8] [9] have demonstrated that the therapeutic efficacy of combining nifedipine and enalapril in treating hypertension with coronary heart disease surpasses that of nifedipine as a stand-alone treatment. However, the existing evidence remains incomplete due to limitations in sample size, warranting further validation. Prolonged administration of high-dose nifedipine may lead to adverse reactions, including the potential risk of. Thus, this article employs a systematic evaluation approach to consolidate and examine the pertinent research both domestically and internationally concerning the effectiveness and safety of combining Tongxinluo with statins in managing hypertension complicated with coronary heart disease. The objective is to provide a solid, evidence-based foundation for clinical use.

## Materials and methods

### Sources of materials and retrieval strategies

A computer-based search was conducted in China Knowledge Net, Wanfang Database, VIP Chinese Science and Technology Journal Database, Pubmed, Webofscience and Cochanelibrary Database from January 2018 to September 2023. The Chinese search formula was: combination nifedipine, enalapril combined nifedipine, coronary heart disease combined with hypertension, and the search terms were connected by AND and expanded by synonyms; the English search formula was: »enalapril AND hypertension AND nifedipine AND coronary heart disease.«

### Inclusion and exclusion criteria

In order to be eligible for inclusion, the study must adhere to the following criteria: (1) The study design should be a randomized controlled clinical trial (RCT); (2) Participants: Individuals diagnosed with hypertension and coronary heart disease according to established standards or guidelines; (3) Intervention: The experimental group was administered a combination of enalapril and nifedipine, whereas the control group received solely nifedipine alongside standard basic treatment. (4) Outcome measures: Primary outcomes included overall effectiveness rate, systolic and diastolic blood pressure, cardiac function, adverse reactions, and ischemic events.

Exclusion criteria: (1) Studies that are not unique or unrelated, including review articles; (2) Controlled trials that are not randomized; (3) Studies without clearly defined diagnostic or efficacy criteria; (4) intervention measures in the trial group were not combined with enalapril and nifedipine, or nifedipine was not used in the control group or nifedipine was combined with other antihypertensive drugs, or other treatment measures in the two groups were different; (5) data were missing, incomplete or obviously wrong.

### Literature screening and data extraction

Two members of the research team performed an extensive examination of existing literature, adhering to specific criteria for inclusion and exclusion. They initially assessed the titles and abstracts of articles, accessing complete texts as necessary. Disagreements were resolved through consultation with external experts. Literature meeting the inclusion criteria was analyzed using a preset table outlining key characteristics, such as total sample size, control group size, test group size, and outcome indicators.

### Literature quality evaluation

We used the revised Jadad scale to evaluate the quality of the included articles. This 7-point scale in volved three questions that assessed randomization, double-blinding, and strategies for addressing withdrawal and loss of follow-up. Studies with a score of 0 were not included in the analysis, whereas those with a score ranging from 1 to 3 were considered as having lower quality. On the other hand, studies scoring bet ween 4 and 7 points were deemed to have higher quality.

### Statistical analysis

In this study, Note Express 3.2 software was used for literature management, while Excel2003 software was employed to collect and extract literature data. Meta-analysis was conducted using Revman 5.4.1 software. The diversity of the extracted data was evaluated by employing the Q test (P value) and the I2 statistic. If the P value exceeded 0.10 or the I2 value was 50% or less, it indicated no heterogeneity, and the fixed effect model (FEM) analysis was utilized. If these conditions were not met, the random effects model (REM) analysis was applied. The results from data pooling were presented using forest plots, displaying the odds ratio (OR) and its corresponding 95% confidence interval (CI). A sensitivity analysis was conducted to assess the consistency of the meta-analysis findings, and publication bias was examined using funnel plots. The significance level was set at =0.05 (two-tailed).

## Results

### Literature search conclusion

**Figure 1:**
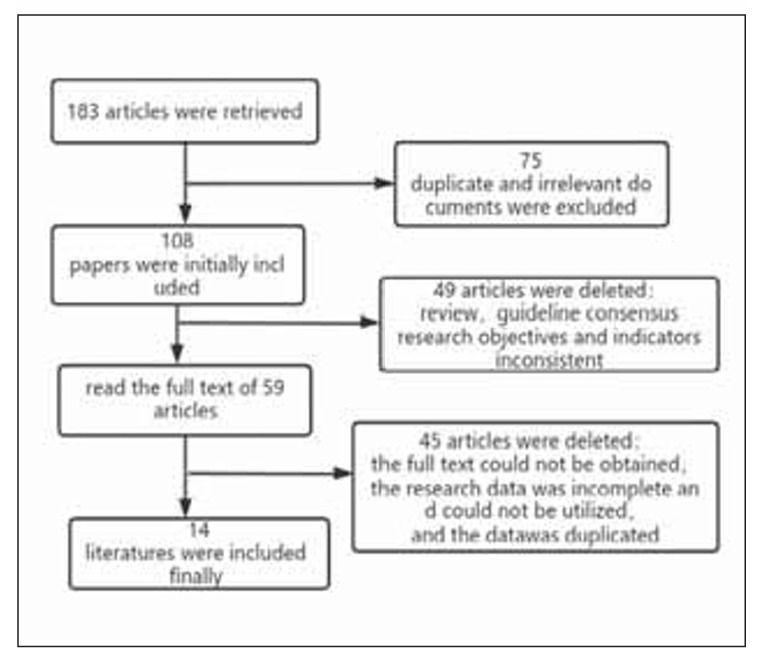
Flow chart of literature screening.

Utilizing the search strategy outlined in the article, a total of 183 pertinent literature sources were initially obtained from databases such as China Knowledge Net, Wanfang Database, VIP Chinese Science and Technology Journal Database, China Biomedical Database, Pubmed, Cochanelibrary, and others. To avoid duplication, duplicate literature sources were removed, resulting in a final selection of 14 literature sources after carefully reviewing their titles, abstracts, and full texts [8] [9] [10] [11] [12] [13] [14] [15] [16] [17] [18] [19] [20] [21]. The process of screening the literature sources is illustrated in Figure 1.

### Literature’s basic characteristics and quality evaluation.

Baseline information typically encompasses factors such as gender, age, duration of illness, prescribed treatment plan, and indicators of outcomes. Detailed description of baseline data is provided in the 14 included literatures. Quality evaluation of the 14 included studies is performed by using the modified Jadad scale. See Table 1.

**Table 1:** Basic characteristics and quality evaluation table of literature.

first author	year of<br>publication	Sample size<br>(cases)		Interventions		outcome measures	Jadad
		test<br>group	control<br>group	test group	control group		
Huang SJ [8]	2023	500	500	Nifedipine+ enalapril	nifedipine	Clinical efficacy, blood pres sure, cardiac function, ischemic events, adverse reac tions	3
Li YF [9]	2022	53	43	nifedipine + enalapril	nifedipine	blood pressure, oxidative stress, adverse reactions, health evaluation	3
Yin HK [10]	2022	58	48	Anticoagulation, antiplatelet, lipid lowering+ nifedipine+enalapril	Anticoagulation, antiplatelet, lipid lowering + nifedipine	blood pressure, cardiac function, adverse reactions	4
Tang M [11]	2021	66	66	dilating coronary+lipid lowering+ Anticoagulation+nife dipine+enalapril	dilating coronary+lipid lowering+ Anticoagulation+ nifedipine	blood pressure, oxidative stress, cardiac function, endothelial dilatation function, adverse reactions	3
Ji MX [12]	2020	67	65	nifedipine+ enalapril	nifedipine	Clinical efficacy, cardiac function, adverse reactions, Lp-PLA2, HCY	3
Zhao N [13]	2022	51	51	nifedipine+ enalapril	nifedipine	Clinical efficacy, blood pressure, adverse reactions, cardiac function	3
Zhu ZX [14]	2023	40	40	nifedipine+ enalapril	nifedipine	blood pressure, cardiac function, Clinical efficacy, adverse reactions, quality of life score	4
Nie X [15]	2023	44	44	dilating coronary+ Anticoagulation+ lipid lowering+ nifedipine+enalapril	dilating coronary+ Anticoagulation+ lipid lowering+ nifedipine	Clinical efficacy, blood pressure, cardiac function, BNP, quality of life score	4
Pan Y [16]	2020	43	43	Anticoagulation+lipid lowering+ dilating coronary + cardioactive+ nifedipine+enalapril	Anticoagulation+ lipid lowering+ dilating coronary +cardioactive+ nifedipine	Clinical efficacy, blood pressure, MDA, SOD, AOPP	3
Qin SP [17]	2018	38	38	nifedipine+enalapril	nifedipine	Clinical efficacy, blood pressure, adverse reactions	3
Li YJ [18]	2019	75	75	nifedipine+enalapril	nifedipine	Clinical efficacy, blood pressure, ischemic events	3
Zeng YL [19]	2018	67	70	nifedipineII+enalapril	nifedipineII	Clinical efficacy, blood pressure, ischemic events	3
Song WQ [20]	2018	18	18	nifedipine+enalapril	nifedipine	Clinical efficacy, blood pressure, ischemic events	4
Ai LN [21]	2023	48	48	nifedipine+enalapril	nifedipine	Clinical efficacy, blood pressure, cardiac function	3

### Results of Meta-analysis

#### Total effective rate

**Figure 2:**
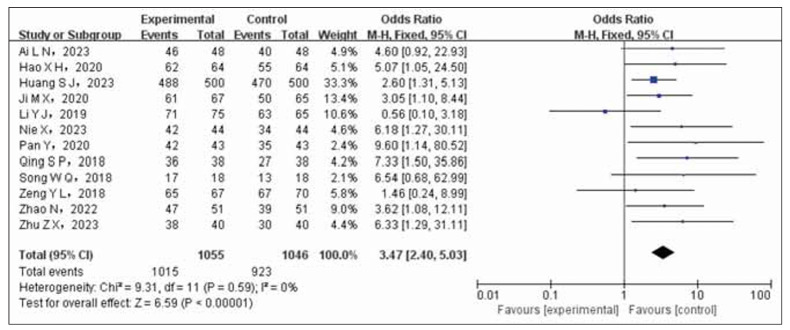
Forest plot comparing the total effective rate of test group and control group.<br>Note: Total effective rate = markedly effective + improved.

Twelve studies, involving a total of 2,101 cases (experimental group: 1,055 cases; control group: 1,046 cases), were examined to compare the overall effectiveness rate. Heterogeneity testing indicated no significant variation among the studies (*p*=0.59, I^2^=0%). Therefore, we employed the Fixed Effects Model (FEM) for data synthesis. The meta-analysis findings demonstrated that combining nifedipine and enalapril in the treatment group resulted in a significantly higher overall effectiveness rate compared to using nifedipine alone in the control group (OR= 3.47, 95%CI (2.40~5.03), *p*<0.00001). Please refer to Figure 2 for more detailed information.

### Changes in blood pressure

#### Changes in systolic blood pressure

**Figure 3:**
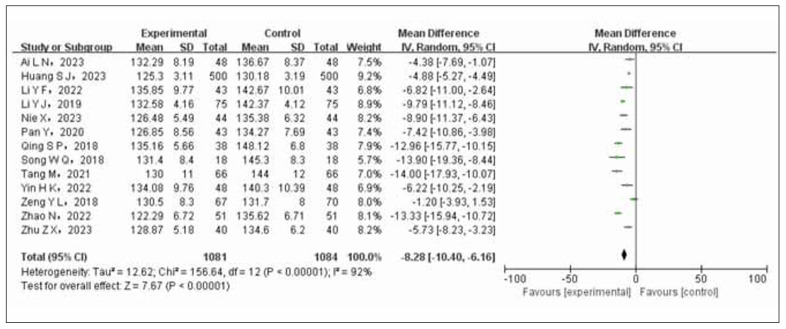
Forest plot of systolic blood pressure comparison between test group and control group.

Thirteen studies were examined, involving 1081 cases in the experimental group and 1084 cases in the control group. The studies exhibited significant statistical heterogeneity (*p*<0.00001, I^2^=92%), necessitating the utilization of a random-effects model for data synthesis. According to the results of the meta-analysis, patients with both coronary heart disease and hypertension who received a combination treatment of nifedipine and enalapril experienced a notably greater decrease in systolic blood pressure compared to those individuals in the control group who were solely treated with nifedipine (OR=-8.28, 95% CI -10.40 to -6.16, *p*<0.00001). Please refer to Figure 3 for a visual representation illustrating these findings.

#### Changes in diastolic blood pressure

**Figure 4:**
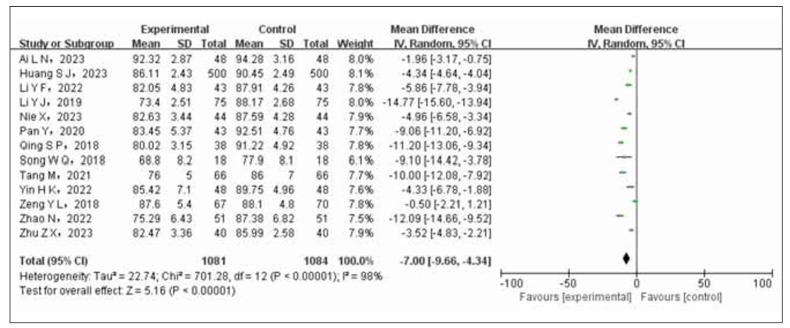
Forest plot of diastolic blood pressure comparison between test group and control group.

Thirteen articles, involving a total of 2165 participants (1081 in the experimental group and 1084 in the control group), were analyzed. Significant statistical heterogeneity (*p*<0.00001, I^2^=98%) among the studies led to the utilization of a random-effects model (REM) for data combination. The meta-analysis demonstrated a notable reduction in diastolic blood pressure among patients with coronary heart disease complicated by hypertension in the trial group (receiving nifedipine combined with enalapril) compared to those in the control group receiving ni fedipine alone (OR=-7.00, 95% CI -9.66 to -4.34, *p*<0.00001). Refer to Figure 4 for a visual representation of these results.

#### Cardiac function

**Figure 5:**
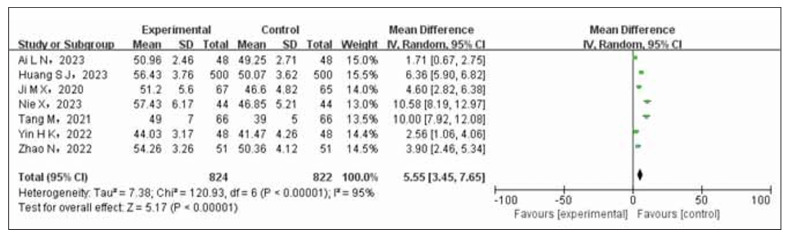
Forest plot of heart function comparison between test group and control group.

Seven studies were conducted to evaluate the impact of a combination treatment of nifedipine and enalapril on cardiac function in patients with coronary heart disease and hypertension. The experimental group consisted of 824 cases, while the control group had 822 cases. The focus was on assessing left ventricular ejection fraction (LVEF). It was observed that there was significant variability among the studies (*p*<0.00001, I^2^=95%). Therefore, a random-effects model (REM) was utilized to combine the data from these studies. The meta-analysis revealed a statistically significant improvement in cardiac function for patients receiving the combination treatment compared to those who received nifedipine alone in the control group. This difference was found to be highly significant (OR=5.55, 95% CI 3.47 to 7.65, *p*< 0.00001), as shown in Figure 5.

### Adverse reactions

**Figure 6:**
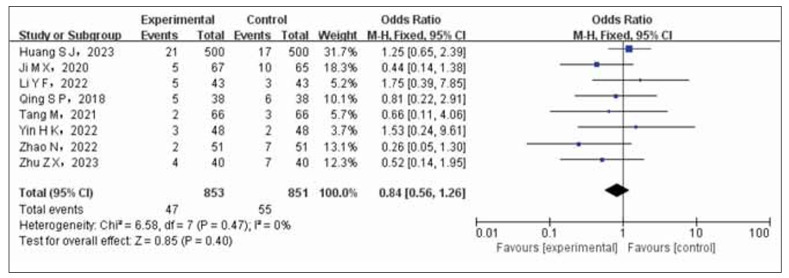
Forest plot of adverse reactions in test group and control group.

The analysis included a total of eight articles, which examined adverse reactions in both the experimental and control groups. The experimental group consisted of 853 cases, while the control group had 851 cases. Statistical tests showed no significant heterogeneity among the studies (*p*=0.47, I^2^=0%), allowing for the use of a fixed-effects model (FEM) to analyze the combined data from these studies. The meta-analysis indicated slightly higher rates of adverse reactions in patients with coronary heart disease and hypertension who received a combination of nifedipine and enalapril compared to those who only received nifedipine in the control group. However, this difference did not reach statistical significance (OR=0.84, 95% CI 0.56 to 1.26, *p*=0.40). Please refer to Figure 6 for a visual representation.

### Incidence of ischemic events

**Figure 7:**
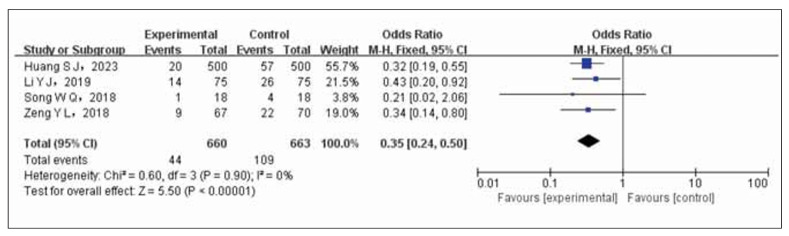
Forest plot of ischemic events in test group and control group.

Four studies, including 660 cases in the experimental group and 663 cases in the comparison group, examined the occurrence of ischemic events after treatment. A test for heterogeneity indicated no significant variation among the studies (*p*=0.90, I^2^=0%), enabling a meta-analysis using the Method of Moments (MEM) on existing literature. The findings demonstrated that when compared to using nifedipine alone in the control group, administering a combination of nifedipine and enalapril was linked to an increased likelihood of ischemic events in patients with coronary heart disease and hypertension. This disparity was statistically significant (OR=0.35, 95% CI 0.24 to 0.50, *p*<0.00001), as illustrated in Figure 7.

### Sensitivity analysis and literature bias check

**Figure 8:**
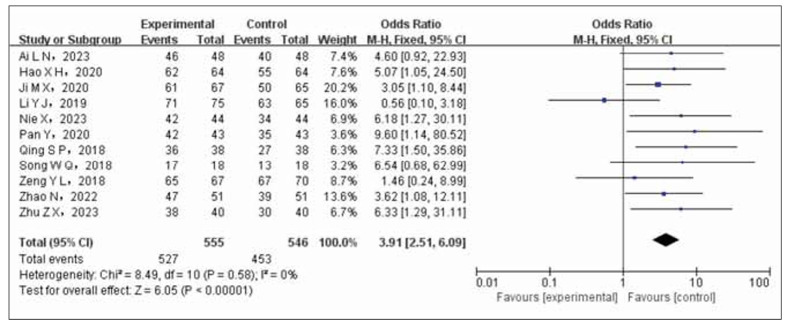
Forest plot for sensitivity analysis.

**Figure 9:**
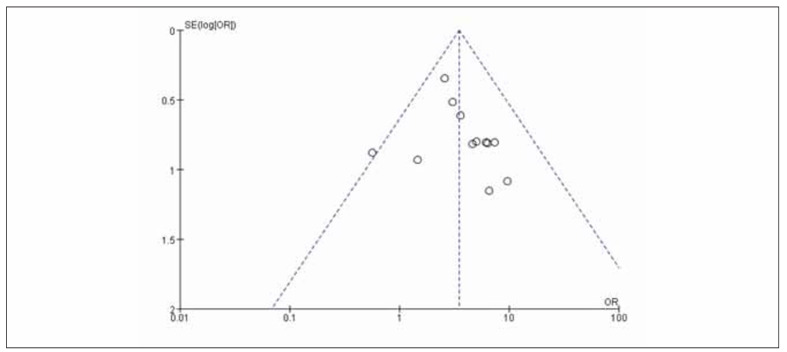
Funnel plot of total clinical effective rate of test group and control group.

The OR (95%CI) was 3.91 after removing the articles with the largest proportion weight and recombining the effect sizes of the articles (2.51~6.09), *p*<0.00001, suggesting that the results obtained in this research are reliable, see Figure 8. Bias test for all outcome indicators involved in this paper shows that funnel plot has asymmetry, indicating bias. Take the total effective rate as an example, see Figure 9.

## Discussion

Hypertension poses a significant risk for the progression of coronary heart disease, and the presence of both conditions in patients makes their situation more complex. Failing to effectively manage blood pressure levels can lead to a worsening of the patient’s condition, heightening the likelihood of adverse cardiovascular and cerebrovascular events, as well as other related complications, which ultimately adversely affects the patient’s prognosis [1]. Recently, the utilization of combination therapy has emerged as a vital treatment strategy in clinical practice, particularly for patients who have both hypertension and coronary heart disease. This approach has demonstrated the ability to ameliorate patient symptoms, optimize blood pressure control, and enhance cardiac function. However, given the limited size of the participant cohorts in these studies, debates persist regarding the effectiveness, safety, and incidence of adverse reactions related to the treatment Thoroughly substantiating these aspects will necessitate further investigations involving larger sample sizes.

The results of the meta-analysis indicated a statistically significant increase in the overall effectiveness rate of the test group compared to the control group (OR=3.47, 95% CI 2.40 to 5.03). The hypotensive effect was more pronounced with a decrease in systolic (OR=-8.28, 95% CI (-10.4~6.16)) and diastolic blood pressure (OR=-7.00, 95%CI (-9.66~-4.34)). There was also a more significant improvement in cardiac function (OR=5.55, 95%CI). However, the control group showed a lower incidence of ischemic events compared to the experimental group (OR=2.86, 95% CI 2.00 to 4.00). A sensitivity analysis confirmed the stability and reliability of the combined effect size outcomes (OR=3.91, 95% CI 2.51 to 6.09, *p*<0.00001).

This study has limitations. The meta-analysis indicated bias, possibly stemming from the extended duration and insufficient sample size of the included literature. Furthermore, the retrieval process was limited to Chinese and English databases, and the selective inclusion of literature in each database could have resulted in sampling bias, which also contributed to bias in the study findings.

## Conclusion

In conclusion, the combination of nifedipine and enalapril demonstrates superior effectiveness in managing hypertension complicated with coronary heart disease when compared to the use of nifedipine in isolation. This finding has significant clinical implications, though it necessitates further validation through future studies that are multi-center, large-scale, and homogeneous in nature.

## Dodatak

### Ethical compliance

Not applicable.

### Author contributions

KW and FS designed the study, WM collected the data, LS analyzed the data, KW and FS prepared the manuscript. All authors read and approved the final manuscript.

### Funding

This study did not receive any funding in any form.

### Conflict of interest statement

All the authors declare that they have no conflict of interest in this work.
